# Keystone Veterinary Medical Association

**Published:** 1891-06

**Authors:** M. W. Drake

**Affiliations:** Secretary


### SOCIETY PROCEEDINGS.

*Keystone Veterinary Medical Association.*—The regular meeting of the Keystone Veterinary Medical Association was held at the College of Physicians, Philadelphia, May 2, 1891.

The President, Dr. W. H. Hoskins, in the chair. The following answered roll call:—Drs. Werntz, Webster, Hoskins, Sellers, Goentner, Weber, Kooker, Lintz and Drake.

The minutes of the previous meeting were read and adopted. Dr. Hoskins, Chairman of the Legislative Committee, said the work was being carried on without much success. Dr. Schreiber failed to appear to read his paper on the subject of Milk Inspection of Philadelphia. Dr. Sellers, said milk and meat inspection should be more rigid in New Jersey as well as in Philadelphia, as he has seen cattle in Camden that came over to Philadelphia to be slaughtered, and the meat used in the manufacture of bolona sausage, that were effected with Tuberculosis and not fit for food.

Dr. Werntz said quantities of diseased meat are brought in from surrounding dairy farms and sold in the markets, we should have a law against it, and the law should by enforced. He was one to introduce a bill before the legislature, in which a veal must be twenty day old, and weighing no less than sixty lbs., before being placed upon the market for food. He says he has seen cattle too sick to walk, brought in after dark, slaughtered and put on our markets.

Dr. Weber says he can drink milk in Philadelphia with perfect safety, due to the present milk inspection of the city. He stated that the officials are examining different samples of milk, under the microscope at the Public Buildings for the Koch Bacillus. Dr. Goentner, said Dr. Weber refused to drink milk at Lancaster when he was there as his guest. Dr. Weber replied, that he did not drink it yet when at home in Lancaster. Dr. Goentner asked, has the Tuberculous Bacillus been found in milk? Dr. Hoskins answered yes! it has been found in milk without any lesion of the glands being detected.

Dr. Kooker thinks milk inspection as it is now being carried on matters little to the Veterinarian, trying to find the Bacillus in milk is not a preventative but simply a scientific investigation. That the Keystone Veterinary Medical Association and State should do more to forward the matter, and that we should have a set of resolutions up before the next legislature, in which the inspection should take place at the farms; he thinks every practitioner should have his local district and be paid for the same, the inspections to take place at certain periods of time. To get these resolutions passed we should go in a body, Physicians, Board of Health and Veterinarians.

He wants resolutions passed to express to the public the views of the Keystone Veterinary Medical Association.

He, as President of the State Association has appointed Dr. Hoskins to read a paper at the next meeting on the subject of Milk and Meat Inspection.

Dr. Sellers thinks water the least harmful of any of the adulterations of milk, Dr. Doremus in his lectures, says, test milk for the sugar, for the sugar seldom varies, the solids vary and the water can be detected. Dr. Weber thinks there should be better Hygenic inspection.

Dr. Hoskins thinks the inspection is clearly from a commercial standpoint, coloring matter was used, also acid to keep it sweet. The present inspection is good but deficient in regard to milk, the work should be carried further, put the resolutions in the medical journals, newspapers, etc.

Dr. Kooker suggests that the president appoint a committee to draw up a set of such resolutions, giving views of this association. Moved and seconded, carried. The president appointed Dr. Huidekoper chairman, Drs. Werntz, Weber, Goentner and Kooker.

Dr. Hoskins cited a case of a green western horse suffering with Influenza, having his tail tied up very tight, the tail swelled to an enormous size which was followed by twenty-three distinct abscesses and a large slough at the end, with a complete relaxation of the sphincter ani, semi membranosis and semi tendinosis muscles, great lameness followed, hardly able to stand, weak pulse, high temperature, is now convalesing, now has a new growth of hair covering the tail.

The vice-president's position being empty, by the resignation of Dr. Ridge, Dr. Werntz was nominated; it was moved and seconded he be elected by acclamation, the chair to cast the vote. Carried

The meeting then adjourned.

M. W. DRAKE, *Secretary.*

